# Design and Implementation of a 2D MIMO OCC System Based on Deep Learning

**DOI:** 10.3390/s23177637

**Published:** 2023-09-03

**Authors:** Ones Sanjerico Sitanggang, Van Linh Nguyen, Huy Nguyen, Radityo Fajar Pamungkas, Muhammad Miftah Faridh, Yeong Min Jang

**Affiliations:** Department of Electronics Engineering, Kookmin University, Seoul 02707, Republic of Korea; ones.sanjerico1@kookmin.ac.kr (O.S.S.); linhnv@kookmin.ac.kr (V.L.N.); ngochuy@kookmin.ac.kr (H.N.); fajar@kookmin.ac.kr (R.F.P.); muhammadmiftahfaridh@kookmin.ac.kr (M.M.F.)

**Keywords:** optical camera communication (OCC), object detection, LED segmentation, YOLOv8

## Abstract

Optical camera communication (OCC) is one of the most promising optical wireless technology communication systems. This technology has a number of benefits compared to radio frequency, including unlimited spectrum, no congestion due to high usage, and low operating costs. OCC operates in order to transmit an optical signal from a light-emitting diode (LED) and receive the signal with a camera. However, identifying, detecting, and extracting data in a complex area with very high mobility is the main challenge in operating the OCC. In this paper, we design and implement a real-time OCC system that can communicate in high mobility conditions, based on You Only Look Once version 8 (YOLOv8). We utilized an LED array that can be identified accurately and has an enhanced data transmission rate due to a greater number of source lights. Our system is validated in a highly mobile environment with camera movement speeds of up to 10 m/s at 2 m, achieving a bit error rate of $10^{-2}$. In addition, this system achieves high accuracy of the LED detection algorithm with mAP0.5 and mAP0.5:0.95 values of 0.995 and 0.8604, respectively. The proposed method has been tested in real time and achieves processing speeds up to 1.25 ms.

## 1. Introduction

The rapid progression of technology has led to the development of innovative technologies that have wide-ranging implications for human existence. The Internet of Things (IoT) is currently regarded as one of the most widely used technologies in recent years [1,2]. The utilization of this technology facilitates the exchange of data and the implementation of integrated control systems, leading to the generation of massive amounts of information and enhanced operational effectiveness across diverse industries. The significance of radio frequency (RF) in the context of wireless data transmission for IoT technologies cannot be neglected [3]. Furthermore, RF technology provides reliable communication channels that give wide coverage and a substantial data rate.

Nevertheless, the utilization of RF spectrum encounters some challenges, such as limited spectrum availability, electromagnetic interference (EMI), multipath reflections that can lead to fading and degrade signal transmission quality, stringent regulatory constraints governing its operation, and vulnerability to unauthorized intrusions [4,5,6]. Furthermore, the health sector has raised concerns regarding the potential health effects of prolonged exposure to RF radiation [7,8,9,10]. As a result, optical wireless communication (OWC) has garnered significant attention as a potential solution due to its advantages, including high-speed transmission, high levels of security, low energy consumption, and high resistance to electromagnetic interference [11,12,13]. LiFi, OCC, visible light communication (VLC), and free-space optical (FSO) communication are considered to be the most promising OWC technologies. Each of these technologies exhibits a distinctive architectural structure, communication protocol, propagation medium, and application scenario.

OCC is a subsystem of OWC that employs a complementary metal-oxide-semiconductor (CMOS) camera as an optical receiver (Rx) to detect signals in visible light (VL) or infrared radiation (IR). In OCC, data are encoded into an invisible frequency-modulated light signal. The CMOS camera on the receiving device captures and decodes the light signal into communication-friendly data. By employing light as the communication medium, OCC offers benefits in terms of data security with minimal risk of cyber-attacks and information leaks [14]. In addition, OCC does not pose a threat to ocular health, as the frequencies of light employed in VLC are generally regarded as safe [15]. The IEEE standard 802.15.7-2018 [16] has been implemented to govern and provide technical guidance for OCC development and interoperability.

In recent years, there has been a notable increase in the use of LED lighting infrastructure across various indoor and outdoor environments in response to their manifold advantages, such as their low energy consumption and robustness. The aforementioned progress has made a major contribution to the increasing capabilities of OCC. Furthermore, a significant amount of research has been conducted on the integration of OCC into automobile technologies. The increasing number of vehicles and roadside units (RSU) has led to the recognition that the importance of OCC will grow, owing to its wide range of applications. These applications consist of platooning, electronic emergency brake lighting, intersection assistance, and forward collision warning [14].

Despite the numerous benefits of the OCC system, there are certain problems that necessitate attention in its development, particularly in regions characterized by high mobility as well as elevated levels of noise and interference. The initial issue emerges in scenarios characterized by high mobility, wherein the occurrence of optical flow on the image sensor can lead to a reduction in the accuracy of LED identification. This, in turn, has a simultaneous impact on the performance of communication [17,18,19]. The second challenge encountered in the field of photography involves the presence of noise originating from the sun, streetlights, and other forms of backlighting [17]. The presence of this cacophony further exacerbates the challenge of identifying the region of interest (RoI). The third challenge lies in the real-time detection and analysis of OCC signals, necessitating extensive computational resources for real-time execution.

In this study, we propose a deep learning-based OCC system combined with an adaptive segmentation algorithm to rectify optical distortions caused by the optical flow on the image sensor. This method seeks to improve the recognition accuracy of LEDs in high-mobility conditions as well as the selection of ROI. Moreover, we optimize the OCC signal processing system so that it can operate in real time with reduced computational costs. This strategy is intended to address the previously mentioned obstacles. To the best of our knowledge, there is no article that applies to You Only Look Once version 8 (YOLOv8) in the OCC system. Our proposed method focuses on LED detection and segmentation in OCC systems based on YOLOv8. The main contributions of the paper are outlined below:

- An effective LED array detection and segmentation based on the YOLOv8 model is proposed to improve the reliability and stability of the OCC system in an environment with high mobility.
- A lightweight LED detection and tracking method that permits the OCC to operate in real time on resource-constrained embedded systems is proposed.
- The proposed method is based on 2D OOK-MIMO, which is compatible with most commercial cameras such as rolling shutter and global shutter cameras. Therefore, this method can be readily implemented using closed circuit television (CCTV), which is widely used and convenient in many locations.
- Finally, the performance of the proposed approach is validated by utilizing the practical implementation of OCC in indoor environments.

This paper’s remaining sections are structured as follows: Section 2 provides an overview of relevant studies for the OCC system and identifies a number of issues. Section 3 provides a detailed explanation of the OCC system architecture proposal. The experimental hardware, software, and evaluation metrics are described in Section 4. Section 5 discusses the results and evaluation of our system’s implementation, including testing the bit error rate (BER) under high-mobility conditions, an aspect that has not been extensively investigated in prior studies. The study concludes in Section 6 with a discussion of potential avenues for future research.

## 2. Literature Review

In recent years, extensive research has been conducted to achieve superior performance in OCC systems, allowing for their application in a variety of disciplines. In their study, Ref. [20] introduced an implementation of the OCC technique which utilizes an indoor positioning system and employs On-Off Keying (OOK) modulation. However, the effective range of communication was limited to a mere 2.7 m, with both the receiving and transmitting devices remaining stationary. The OCC system was developed and deployed using LabView, as documented by [21]. However, the utilization of LabView in compact, cost-effective modules such as the Jetson Nano Kit poses certain implementation difficulties.

Another study [22] investigated the phenomenon of blurring in optical vehicle communication systems, including motion blur and blurring induced by rainy, foggy, or snowy weather conditions, in an effort to improve OCC performance in a high-mobility environment. The presence of other light sources has the potential to induce a rise in the bit error rate (BER) of the system as a result of the interference they generate at the receiver. The author proposed a neural network (NN)-based method for performing RoI selection and detecting the target transmitter on the receiver of a VLC or OCC system to mitigate the impact of parasitic light incidents. The author of a research study [23] proposed the utilization of OCC-based convolutional neural networks (CNNs) for the purpose of detecting LEDs within the context of the Internet of Vehicles (IoV). Nevertheless, the implementation of OCC systems developed in Matlab is a significant challenge when applied to devices that have limited computational resources. Furthermore, the precision of the detected LEDs is only 60%.

In order to tackle the issue of accuracy and real-time implementation, the researchers utilized the YOLOv2 model for the purpose of identifying RoI. This model has been proven to reach the highest level of accuracy while maintaining real-time performance, as stated in the study [24]. The proposed methodology effectively produces ROI in systems, demonstrating a significantly high processing frame rate and satisfactory detection accuracy. However, the experimental findings pertaining to LED recognition indicate that the transmission distance is limited to a mere range of 0.5 to 1 m.

Nguyen et al. [25] proposed a MIMO C-OOK scheme based on rolling-shutter effect for the mobility environment using a deep learning network. This method achieved a communication distance of up to 22 m with a considerably low bit error rate when considering the movement speeds at 2 m/s. However, the proposed method only works on the rolling-shutter camera which limits its implementation.

Guan et al. [26] proposed the utilization of CNN within the realm of VLC to enhance the precision of classifying Optical Fringe Codes (OFC) to a level surpassing 95%. Nonetheless, this approach necessitates that the RGB-LED and camera be aligned in parallel. Further advancements in VLC and OCC technologies are needed to enhance the transmission range and facilitate LED identification at different angles and distances in IoT applications.

Although significant progress has been made, experimental demonstrations of the efficacy of an OCC system in high-mobility environments are limited. In addition, there is a dearth of research into lightweight deep-learning models for OCC that can improve mobility, communication distance, and resilience against optical noise sources. Consequently, the objective of this study is to present an OCC system that incorporates YOLOv8 for LED detection and tracking on the receiver side. YOLOv8 [27] is designed to be a lightweight, quick, accurate, and precise model, making it the ideal option for detecting and tracking LEDs in the OCC system. By integrating the YOLOv8 model into the OCC system, our suggested approach offers enhanced mobility, precise detection capabilities, and real-time performance on resource-constrained devices.

## 3. Description of the Proposed System Architecture

**Figure 1:**
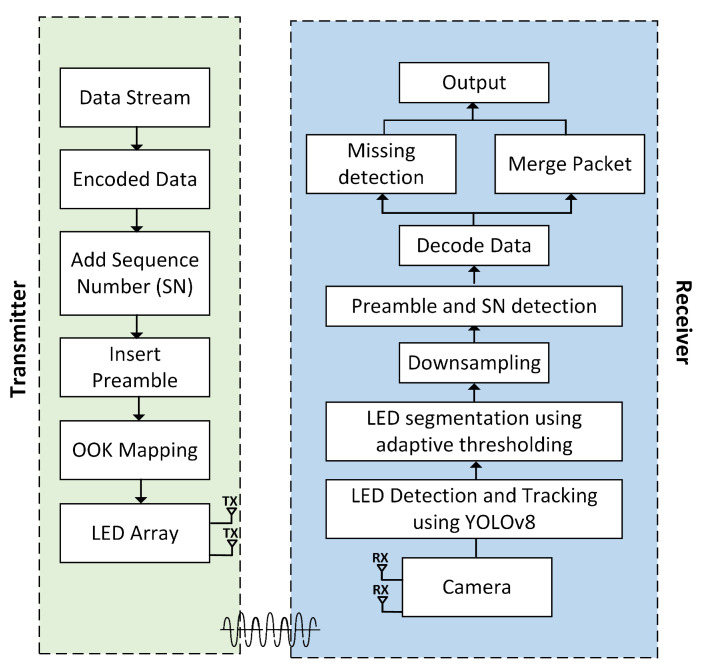
Reference architecture of OCC for a system-based 2D OOK-MIMO and deep learning technique using an LED array.

This section provides a description of the deep learning-based two-dimensional on-off keying multiple-input multiple-output (2D OOK-MIMO) OCC. The architecture of a 2D OOK-MIMO transceiver that incorporates a deep learning system is illustrated in Figure 1. The transmitter encodes the data information, and the fundamental principle of the OCC system is to calibrate the optical signal intensity for dependable data transmission and reception [21]. The enhancement of the security and precision of the communication system can be achieved through the utilization of a promising modulation scheme. The primary form of amplitude-shift keying modulation that serves as the foundation is the OOK scheme. This scheme utilizes two distinct data statuses, namely, “ON” and “OFF”, which are symbolized by the binary digits “1” and “0”, respectively.

Compared to the OOK described in [21,28,29], we detected LEDs using YOLOv8 algorithms. By positioning each LED in an LED array according to the spatial frame format of our proposed scheme, which is based on image processing techniques, the receiver side can readily detect and resolve data code.

### 3.1. Transmitter

In order to ensure reliable data transmission across a network, it is necessary to optimize the payload of the data for transmission. The optimization technique commonly involves the encoding of data into a format that is understandable by computers. The process of encoding data involves converting information into digital signals, which are represented by binary numbers, specifically “0” and “1”. The encoding procedure entails mapping these numerals to digital signals transmitted over a communication channel using various patterns of voltage or current levels. LEDs are employed for the purpose of visually representing digital signals during the transmission process. In the context of this system, the representation of a binary digit “0” is achieved by deactivating the corresponding LED, and a binary digit “1” is displayed by activating the LED. The utilization of this encoding process is vital to ensuring precise and reliable data transmission over a network. The utilization of digital signals enables enhanced resistance against noise and various forms of interference, thereby increasing the likelihood that transmitted data will be received accurately.

The subsequent phase involves the addition of the sequence number (SN). The SN is employed within a communication system to distinguish between the received data packets at the receiver. Moreover, the SN allows the cameras on the receiving side to identify missing packets in the event of oversampling. The SN designates the payload and contains sequence information for every data packet. The duration of the SN can be modified in accordance with the conditions and requirements of the system. Channel conditions determine the SN length, but it is possible to shorten it to improve system performance. The 2D MIMO technique was implemented in this research using an SN length of 2 bits. The decision to use this specific length for the SN was made by considering the trade-off between system performance and computational complexity.

The preamble, which is the first segment of the symbol frame, serves the essential purpose of synchronizing and identifying the start of a frame. This enables the receiver to decode the data efficiently. The presence of any fault in the preamble section of a frame results in the transmission of a defective bit, thereby making the data unobtainable by the receiver. On the other hand, a well-constructed preamble enables the extraction of information contained within the frame, thereby enhancing the accuracy and reliability of transmission. This preamble facilitates the straightforward detection of image frame initiation.

### 3.2. Receiver

**Figure 2:**
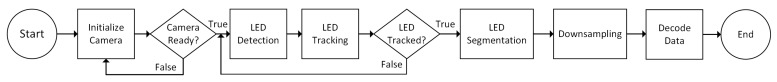
Flowchart for the processing algorithm at each frame on the receiving side.

In the context of the OCC system, the dependable and real-time identification of LED arrays in complex environments remains an open issue. In order to tackle this matter, we suggest the utilization of the YOLOv8 model, which demonstrates both feasibility and effectiveness in the identification and tracking of LED. This particular model demonstrates the capability to perform inference at high speed while maintaining a high level of precision in detection. The flowchart depicted in Figure 2 illustrates the processing technique executed on every frame throughout the receiving process. The OpenCV library is employed for camera initialization and activation. Upon successful camera initialization, the YOLOv8 model is utilized to facilitate real-time detection and tracking of an LED array, and this process continues until the LEDs are successfully detected. Following successful LED detection, segmentation is carried out using adaptive thresholding. Subsequently, downsampling is performed to determine the ON/OFF status of the LEDs. This process involves placing the center points of the LED array and calculating the distances between the LED array to ascertain their individual positions. This step is important for preamble detection and SN detection. Furthermore, the process of data decoding is executed independently for each image frame. Each frame is decoded separately to ensure fast and responsive data processing, with the goal of minimizing latency in each processed image frame. During this stage, data from each frame are transformed from a bit format into a format readable by the user. The processes of missing detection and packet merging are conducted in parallel to enhance the quality of received data and reduce communication latency. Upon the successful completion of all processes, the output results are displayed on a monitor.

The YOLO technique, renowned for its object identification capabilities, is widely recognized in the field of deep learning [30]. The YOLO model differs from traditional object identification methods by utilizing a convolutional neural network to directly predict the bounding box and corresponding probabilistic confidence of visual objects. This approach leads to a notable enhancement in detection precision [31]. Currently, the YOLO architecture encompasses a total of eight variations, namely, YOLOv1 through YOLOv8. Based on prior research studies [32], it has been observed that YOLOv1 encounters challenges pertaining to its ability to generalize, perform accurate detection, and recognize small objects. Subsequently, Redmon et al. [33,34] introduced YOLOv2 and YOLOv3 as successive advancements in response to the aforementioned concerns. These iterations aimed to improve accuracy by using multi-scale detection and layer-merging techniques. Furthermore, the introduction of YOLOv4 has yielded noteworthy outcomes, demonstrating a substantial improvement over YOLOv3 in terms of both processing speed and average accuracy [35]. Following the release of the YOLOv5 [23] and YOLOv6 [36] models, it was observed that YOLOv5 exhibited superior average speed compared to YOLOv4 and YOLOv6. Conversely, YOLOv6 indicated superiority in terms of average accuracy. Subsequently, the introduction of YOLOv7 [37] aimed to enhance accuracy while maintaining inference speed and reducing training time. The YOLOv8 model has gained recognition because of its remarkable speed in detecting objects, with a detection rate of 280 frames per second [38]. The significant improvements achieved by YOLOv8, such as in terms of speed and accuracy, have a positive impact on the results of LED detection and segmentation. The performance of this model enables the overcoming of challenges in accurately and efficiently identifying and separating LED areas, particularly in the context of real-time communication. Through the implementation of novel convolutional techniques and enhancements in data mosaics, YOLOv8 provides a valuable contribution to addressing the need for reliable and responsive object detection.

**Figure 3:**
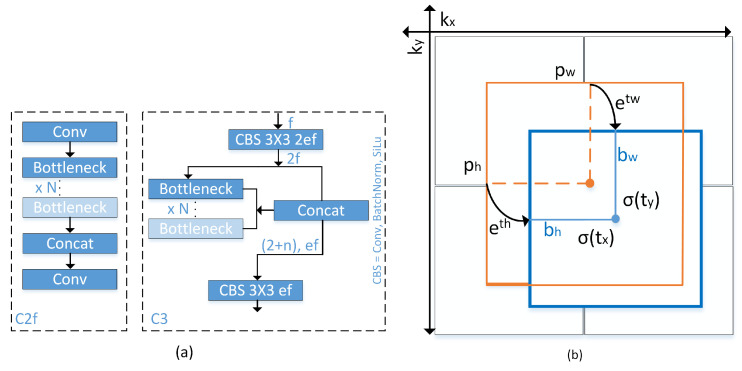
(**a**) Replacement of C2f module with C3 module in YOLOv8; (**b**) Visualization of an anchor box in previous YOLO version.

The YOLOv8 model introduces novel convolutional techniques, anchor-free detection, and mosaic data improvement [38,39,40]. In Figure 3a, a novel 3 × 3 convolution operation is executed, resulting in the replacement of C2f with C3. In the C2f model, the aggregated outputs of all bottleneck layers are employed, but in the C3 model, just the output from the final bottleneck layer is utilized. This approach leads to a reduction in the overall number of parameters and the size of the tensor, hence enhancing the efficiency of convolutional operations employed in the model.

Previous YOLO version implemented anchor box method, as illustrated in Figure 3b and can be formulated as follows:

(1) $$b_{x}=\sigma \left(t_{x}\right)+k_{x}$$

(2) $$b_{y}=\sigma \left(t_{y}\right)+k_{y}$$

(3) $$b_{w}=p_{w}\times e^{w}$$

(4) $$b_{h}=p_{h}\times e^{h}$$

where the variables $b_{x}$, $b_{y}$, $b_{w}$, and $b_{h}$ represent the centroid, width, and height of the predicted Bounding Box (BB). Additionally, the variables $t_{x}$, $t_{y}$, $t_{w}$, and $t_{h}$ denote the output of a neural network. Furthermore, the variables $k_{x}$ and $k_{y}$ represent the coordinates of the upper left corner of the anchor box, and $p_{w}$ and $p_{h}$ denote the width and height of said anchor box. $e^{w}$ and $e^{h}$ represent a logarithmic space transformation.

Unlike previous YOLO, the YOLOv8 introduces anchor-free detection that can directly estimate the centroid of the object, resulting in a reduction in the number of square predictions. The utilization of this technique has the potential to accelerate the process of Non-Maximum Suppression (NMS), thereby improving the mean Average Precision (mAP) of object detection. Previous models in the field of YOLO utilized a complex anchor box mechanism, which led to a decrease in processing speed for object centre detection and a logarithmic space transformation is employed to refine the precise height and width of the bounding box. In this particular case, YOLOv8 utilizes the sigmoid function to generate ($t_{x}$, $t_{y}$), which represents the centre bounding box coordinates ($b_{x}$, $b_{y}$).

Furthermore, a novel approach called the mosaic data enhancement technique was developed, wherein many items are merged into a single entity and afterwards utilized as input for the model. This strategy facilitates the model’s ability to identify and analyze different item placements, resulting in a decrease in the required training time for the given dataset.

The YOLOv8 architecture includes four depth-specific variants: YOLOv8s, YOLOv8m, YOLOv8l, and YOLOv8x. Specifically, variants with a deeper backbone network produce more feature maps, but at the cost of increased computational complexity. In this study, we analyzed and selected the structure with the smallest weight in order to ensure LED detection and tracking accuracy standards and device compatibility.

LED segmentation is the subsequent stage. The conventional threshold value approach, which utilizes one or more thresholds, is a simple image segmentation method. Basic thresholding replaces pixel values that are greater than, equal to, or smaller than the threshold *T* value. However, this approach is a global thresholding technique, which means that the same value of *T* is used to evaluate and classify all pixels in the input image as foreground or background. The issue is that a single *T* value may not be adequate in this situation. One value of *T* may work for one segment of the input image but fail catastrophically for another due to variations in lighting conditions, shadowing, and other factors. Instead of training a deep neural segmentation network such as CNN, Mask R-CNN, or U-Net, in this study, LED segmentation was performed using adaptive thresholding. We autonomously segmented our foreground and background using adaptive thresholding.

**Figure 4:**
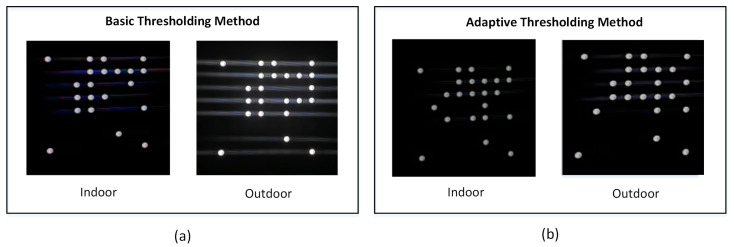
LED segmentation based on (**a**) Basic thresholding method and (**b**) Adaptive thresholding.

Adaptive thresholding evaluates a small group of nearby pixels at a time, computes the *T* for that particular local region, and then segments the image. The size of the pixel neighbourhood must be large enough to encompass both background and foreground pixels for local thresholding to be effective. We frequently discover that a variety of neighbourhood sizes produce acceptable outcomes. We are not necessarily pursuing the optimal *T* value that will determine the success of our thresholding output. In the arithmetic mean, each neighbourhood pixel contributes equally to the calculation of *T*. In contrast, the pixel values further from the centre of the region’s coordinate system have less of an effect on the overall calculation of *T* in the Gaussian mean. The general formula for calculating *T* is [41]:

(5) $$T=mean(I_{L})-C$$

where the mean is either the arithmetic or Gaussian mean, $I_{L}$ is the image’s local subregion *I*, and *C* is a constant that can be used to adjust the threshold value *T*. In order to determine the value of the constant “*C*” in adaptive thresholding, an initial estimation of the constant “*C*” is conducted, either from zero or estimated from the image characteristics. Subsequently, apply adaptive thresholding to the image, followed by evaluating the segmentation results for false positives and false negatives. Adjust the value of “*C*” iteratively; decrease it if the threshold is overly strict (false negatives) or increase it if there are excessive details (false positives). Validate the chosen value across multiple images to obtain the optimal constant “*C*” for optimal segmentation outcomes. The utilization of adaptive thresholding techniques for segmentation purposes is considered to be more advantageous compared to global thresholding techniques. Moreover, LED segmentation based on the adaptive thresholding method can bypass the time-consuming and computationally costly process of training a dedicated CNN, Mask R-CNN, or U-Net segmentation network. Figure 4 illustrates a comprehensive comparison between LED segmentation techniques utilizing simple thresholding and adaptive thresholding methods. Figure 4a illustrates the basic thresholding performance in both indoor and outdoor settings. It is obvious that in the context of outdoor settings, the implementation of basic thresholding resulted in a significant amount of noise, indicating that this particular approach does not exhibit optimal performance across all conditions. In contrast, the LED segmentation technique utilizing adaptive thresholding, as depicted in Figure 4b, exhibits superior performance across all environmental conditions.

**Figure 5:**
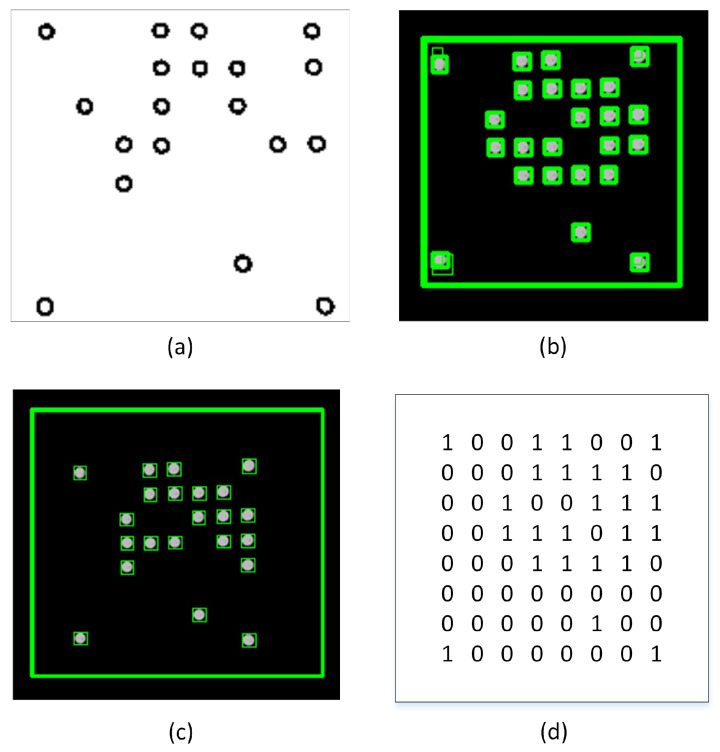
(**a**) LED segmentation based on adaptive thresholding method; (**b**) Recognition of contours before filter is used; (**c**) Recognition of contours LED after filter is used; (**d**) Raw digital data before downsampling.

In addition, we employed a method of image noise filtering based on contouring and size algorithms in order to reduce any noise that may have occurred. A contour refers to a continuous line that connects various points along a border, wherein these points have an identical colour or intensity value. Contours play a crucial role in the determination of the geometry of LEDs. After the implementation of adaptive thresholding on the binary image, the extraction of the contours of the LEDs becomes a straightforward task. Contour separation is a preprocessing methodology commonly employed in digital image analysis to extract and isolate relevant information pertaining to the overall contour of the image. The aim of this study is to distinguish the various features of the contour, which can then be utilized for the purpose of classifying the sample. By identifying the contour precisely, it is possible to obtain precise characteristic samples that facilitate the identification of LEDs. To attain the desired LED configuration, a Gaussian filter is employed on the edges of the contours. After implementing an adaptive threshold, the resulting image resembles Figure 5a. Following this, the contours are detected, as depicted in Figure 5b. Nevertheless, a few of these outlines are not appropriate for our requirements. Consequently, a filter is implemented on the edges of the contours to generate contours around the LEDs and remove the noise, as depicted in Figure 5c. Ultimately, the unprocessed digital data can be readily obtained by utilizing the LED outlines, as illustrated in Figure 5d.

**Figure 6:**
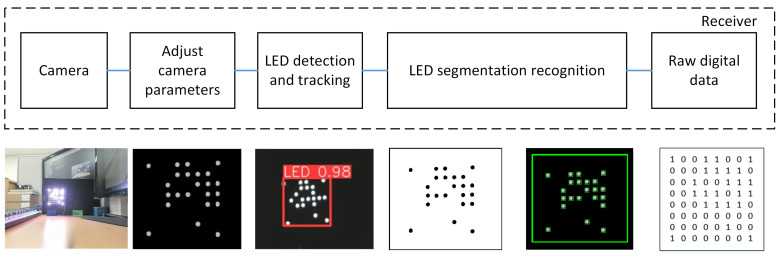
Receiving-side 2D MIMO processing algorithm.

After the acquisition of the raw digital data, it becomes necessary to rapidly identify and assess the preamble pattern. Through the identification of the preamble pattern, the system possesses the capability to assess the authenticity and reliability of the provided raw digital data. Therefore, the original digital data extracted from a frame may be either precise or imprecise. Moreover, in cases where the packet data are precise, the process of SN identification is employed to distinguish between individual packets. The ensuing stages involve decoding and comprehensive data collection. The whole structure of the receiver-side processing approach is illustrated in Figure 6. On the other hand, in cases where the data are imprecise, the receiver will assess the next raw digital data.

## 4. Experimental Setup

### 4.1. Data Collection and Labelling

**Figure 7:**
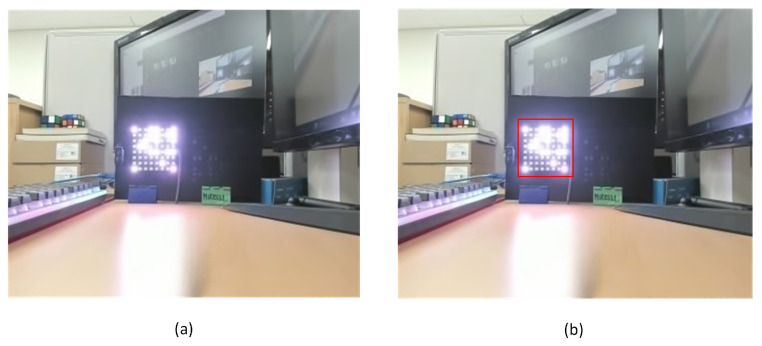
(**a**) Sample of raw data before labelling; (**b**) After labelling process.

In order to improve the accuracy of the LED identification system, it is important to gather and annotate image data prior to the training of the YOLOv8 model. In order to accomplish this objective, the labelling software was employed to annotate picture labels, thereby facilitating image processing. A dataset including 5000 images has been obtained and annotated, with 4000 images allocated for the training set and 1000 images designated for the validation set. The sample data before and after the labelling process are illustrated in Figure 7.

### 4.2. Hardware Setup

In this paper, a variety of distances and velocities were employed to evaluate the efficacy of our model. Our goal was to ensure that our model is suitable for use in highly mobile scenarios, such as vehicle-to-vehicle (V2V) systems that employ the OCC technique and can cover long distances. To accomplish this objective, we varied the speed with which we moved the camera receiver over a predetermined distance during each test. Then, we calculated the BER for each velocity value, allowing us to assess the system’s reliability under conditions of high mobility. In addition, we assessed the efficacy of the system by evaluating the precision and execution time of the LED detection model for each frame of the deep learning algorithm.

**Figure 8:**
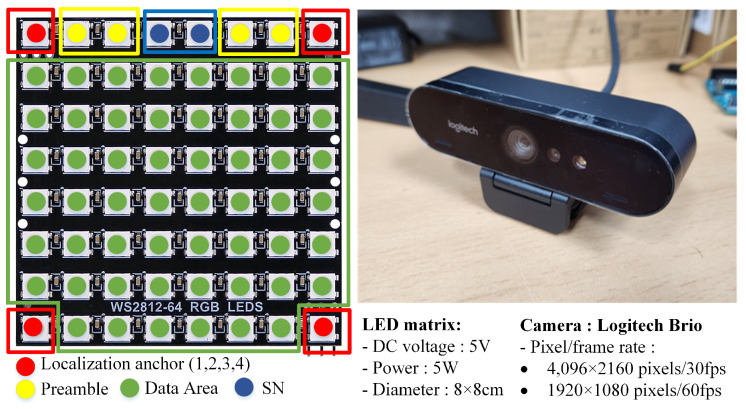
The overall process in the receiver-side with the sample images.

Figure 8 depicts the 8 × 8 rectangle LED matrix on the transmitter side and the Brio UHD Pro Camera, manufactured by Logitech International S.A. in Lausanne, Switzerland, on the receiver side. In addition, our system contains sensors, such as the DHT22 sensor manufactured by ASAIR in Guangzhou, China for measuring temperature, humidity, and vibration and the TFmini S LiDAR sensor produced by Benewake in Beijing, China for measuring the distance between two objects in real-time; the output can change based on environmental conditions. In addition, our system can be applied to a variety of applications, such as the V2V system, where the transmitted data can include, among other vehicle-related information, speed and travel direction.

The four corners of the LED that were most distant served as corner detection anchors. From these corner coordinates, it was straightforward to calculate the coordinates of all LEDs using a perspective transformation. The system transmitted data using 54 LEDs, including the SN bits.

### 4.3. YOLOv8 Training Setup

The input image has a resolution of 1920 × 1080 pixels, the number of training epochs is set to 1000, the initial learning rate is 0.001, the dropout rate is 0.2, and the decay callback will decrease the learning rate by multiplying it by 0.01 if the loss validation is not decreased in five epochs, and the early stopping strategy was implemented to prevent overfitting. Then, we set the batch sizes to 32, 16, and 8, respectively, and compared the efficacy of each batch size. All experiments were trained using the PyTorch framework and executed in Python 3.10.0. an Intel^®^ Xeon^®^ Silver 4215R processor, an NVIDIA RTX 3090 graphics processing unit, and 24 GB of RAM were employed to perform this task.

### 4.4. Evaluation Metrics

The performance of the LED detection network is evaluated using the following metrics:

(6) $$\mathrm{IoU}=\frac{B\cap B_{gt}}{B\cup B_{gt}}$$

(7) $$\mathrm{Average}\;\mathrm{IoU}=\frac{\sum_{i=1}^{N}IoU_{i}}{N}$$

(8) $$\mathrm{Precision}=\frac{\mathrm{TP}}{\mathrm{FP}\;+\;\mathrm{TP}}$$

(9) $$\mathrm{Recall}=\frac{\mathrm{TP}}{\mathrm{FN}\;+\;\mathrm{TP}}$$

where *B* is the predicted box, $B_{gt}$ represents the ground truth box, and *N* represents the total number of objects in the images.

The IoU value will thereafter be employed to determine the true positive (TP), false positive (FP), true negative (TN), and false negative (FN) for each prediction. The detection box is assigned TP if the IoU between it and the LED bounding box is greater than 0.5. In all instances other than the one being discussed, the detection box is designated as an FP. In cases where the LED bounding box does not have a corresponding detecting box, it is indicated as an FN. The terms TP and FP refer to the accurate and inaccurate identification of LEDs, respectively, while FN indicates the count of LEDs that were not identified. The inclusion of TN is unnecessary for the purposes of this binary classification task, as the foreground is consistently maintained for LED detection. The Average IoU in Equation (7) is computed by summing the IoU values for each object or segment and then taking their average. A higher Average IoU value indicates better model performance in accurately recognizing and delineating objects.

The precision rate refers to the ratio of accurately predicted positive instances by the YOLOv8 model. On the contrary, the recall rate pertains to the ratio of correctly identified true positives by the model within the provided dataset. The explicit evaluation of detection accuracy is hindered by the relationship between precision and recall rates. Hence, in order to quantify the reliability of LED detection, the AP was incorporated into Equation (10). The quantity AP represents the integral of the precision and recall values over the whole range of the precision-recall curve. Furthermore, the AP denotes the average accuracy rate of detection, which is measured on a scale ranging from 0 to 1. The expression is articulated in the following manner:

(10) $$\mathrm{AP}=\int_{0}^{1}Pr\left(Re\right)dRe$$

(11) $$\mathrm{mAP}=\frac{1}{n}\sum_{k=1}^{k+n}AP_{k}$$

In Equation (11), mAP is used to characterise a model’s overall quality. The mAP is computed by averaging the AP for each class. mAP takes into consideration the trade-off between precision and recall and the number of FP and FN. Therefore, this property is suitable for the majority of detection applications.

## 5. Result and Discussions

### 5.1. YOLO Train Result

In this section, we will compare YOLOv8s as the chosen model to other algorithms in the YOLOv8 family, such as YOLOv8m, YOLOv8l, and YOLOv8x, in order to assess the performance of the chosen LED detection model. This study assesses the precision, speed, GFLOPs, and weight of the most recent detection algorithms. Based on the evaluation indicators described in this paper, Table 1 compares the state-of-the-art LED detection algorithms with the proposed YOLOv8s.

Table 1 provides a summary of the LED detection model proposed in this investigation, based on YOLOv8. In terms of mAP0.5, mAP0.5:0.95, and precision performance, our analysis indicates that there is no significant difference between the YOLOv8s, YOLOv8m, YOLOv8l, and YOLOv8x models. The YOLOv8x achieved the highest overall performance with a slight difference. However, there is a positive correlation between processing time, GFLOPs, and the weight of the model, whereas larger GFLOPs and heavier weight will increase processing time significantly. In contrast, the batch size has a negative correlation with the processing time, where a smaller batch size will increase the processing time. Furthermore, the YOLOv8s with batch size 32 outperformed all the other models in terms of processing time, weight, and GFLOPs values of 1.25 ms, 22.5 MB, and 28.65, respectively, with comparable mAP and precision.

Table 2 compares the performance of the YOLOv8 detection algorithm with YOLOv5. In this comparison, we set the batch size to 32 and the number of training epochs to 1000, maintaining the same specifications. According to the literature [39], the performance of both models is said to be very similar. Therefore, we conducted a retest based on that literature. Although the performance of both YOLO types is said to be very similar, we found significant differences in terms of processing time and the size of the weights produced by each YOLO. We found that YOLOv8 performs better compared to YOLOv5, with a processing time of 1.25 ms and a weight size of 22.5 MB. Therefore, YOLOv8s is the most suitable model to implement on resource-constrained devices in terms of processing performance and portability.

**Figure 9:**
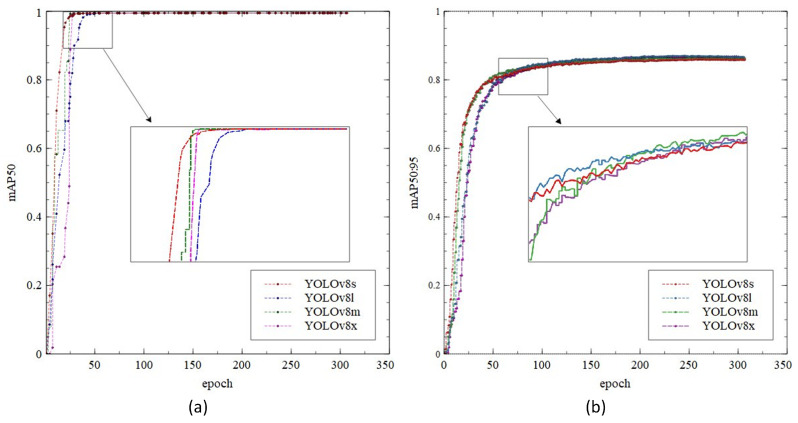
The LED training of (**a**) mAP0.5; (**b**) mAP0.5:0.95.

Figure 9 depicts the comparison of the YOLOv8 training process in terms of mAP0.5 and mAP0.5:0.95. Figure 9a shows the training process over 300 epochs and saturated after approximately 75 epochs. Furthermore, even though all the models achieve similar mAP results, YOLOv8s convergence is slightly faster than the other model. On the other hand, Figure 9b depicts a lower mAP compared to Figure 9a, with a maximum value of 0.86964. From the figure, we can see that the model’s convergence is slower and saturated after 100 epochs.

### 5.2. BER Estimation

In this paper, we have developed comparative experiments to evaluate the system’s performance. The term BER refers to the proportion of bit errors that occur within a given period of time expressed as a ratio between the number of bit errors and the total number of bits transmitted. A bit error occurs when a data payload received via a transmission channel experiences changes in its bits as a result of noise, interference, distortion, or transmission errors caused by movement.

**Figure 10:**
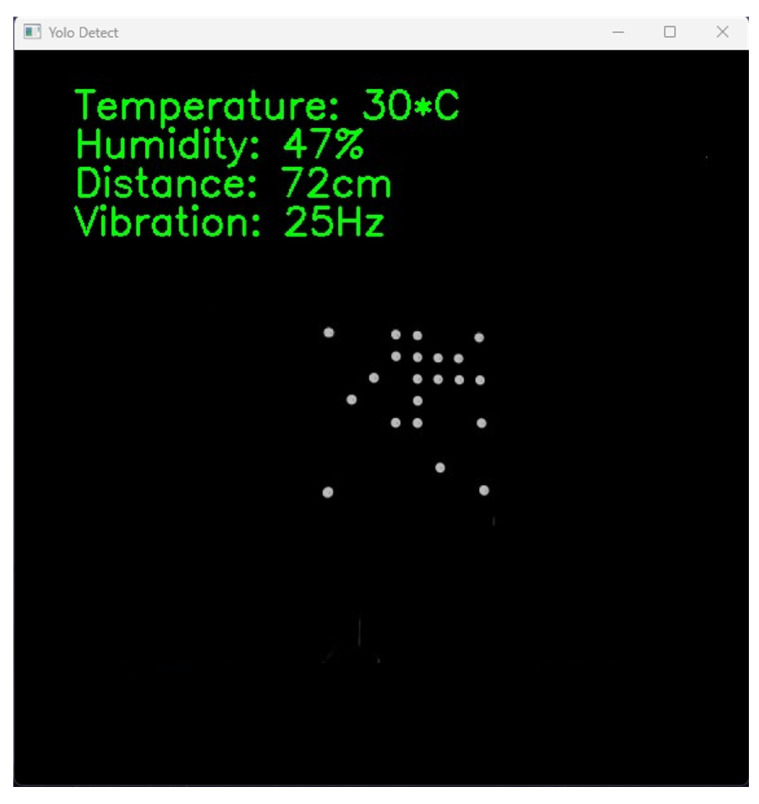
The received interface is displayed on the screen.

Figure 10 displays the successful OCC experimental results based on our proposed method with no bit error. In this implementation, we adopted the Logitech Brio camera with a resolution of 1920 × 1080 pixels and a frame rate of 60 fps. Our system is capable of achieving data transmission speeds of up to 3.84 kbps when using an 8 × 8 LED array configuration, and this data rate can increase up to 15.36 kbps when utilizing a 16 × 16 LED array configuration. However, in our implementation, we focused on employing an 8 × 8 LED array.

**Figure 11:**
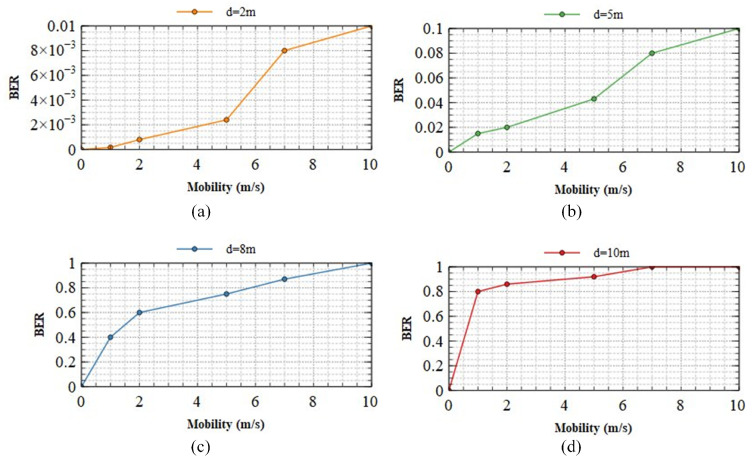
The BER performance of our scheme at different communication distances with changes in the speed of the camera (**a**) 2 m; (**b**) 5 m; (**c**) 8 m; (**d**) 10 m.

Figure 11 displays the BER performance at varying communication distances with changes in camera velocity at (a) 2 m; (b) 5 m; (c) 8 m; and (d) 10 m. In our implementation, we systematically move the camera and LED array congruently in the same directions (up, down, left, and right) at velocities ranging between 0–10 m/s. This approach is adopted to comprehensively assess the system’s performance. The alignment of the camera and LEDs in terms of direction and velocity ensures that communication distances remain consistent even during dynamic detector movement, facilitating the calculation of BER under stable conditions. At all distances, when the transmission system is stationary, the bit error rate (BER) remains at zero, permitting the system to be deployed. At 2 m, Figure 11a demonstrates that the transmitted motion has a minimal effect on the BER. Even at 10 m/s, the BER does not exceed $10^{-2}$. In this scenario, the system demonstrates outstanding mobility support, and its performance remains consistent despite a variety of mobile circumstances. Therefore, faultless transmission is possible within a 2 m range, regardless of system motion. Figure 11b shows that between 5 m and 8 m, the system’s mobility begins to affect transmission. The BER of $10^{-1}$ indicates that the system’s performance is unaffected by its mobility, even at 10 m/s, as demonstrated by the system’s performance. Regarding Figure 11c,d, it can be seen that the mobility of the system has a substantial impact on the BER. Therefore, ensuring the system’s optimal performance over extended distances and under conditions of high mobility becomes impossible. Improving the performance of OCC systems over extended distances and in scenarios with high mobility represents a significant challenge for the future.

## 6. Conclusions

In this paper, we propose an LED detection, tracking, and segmentation system based on YOLOv8s in conjunction with the conventional image processing method used in the OCC system. Specifically, we proposed the YOLOv8s model, which is a viable and effective model for LED detection due to its ability to function in real time while preserving detection precision. In addition, our OCC system’s combination of object detection and tracking aids in reducing the system’s processing time.

Therefore, our system is capable of real-time operation on embedded devices with limited configurations. Furthermore, we propose a method for LED segmentation recognition based on conventional image processing which reduces processing complexity compared to deep neural network algorithms while maintaining a high level of precision. Additionally, we have implemented a system to evaluate the performance of the OCC system at different transmission ranges in mobile environments. The results demonstrate that our OCC system exhibits high efficacy at a range of 10 m in a low-mobility environment and achieves high mobility (speed = 10 m/s) at a range of 2 m. However, obstacles remain when the system operates with high mobility over long distances. Our design can be utilized in future OCC systems that require a high degree of mobility.

In the future, further research on OCC can explore the utilization of lasers as the transmitter to increase the transmission distance and investigate novel modulation schemes to enhance the data rate of this system. Additionally, developing a cloud-edge architecture with a lightweight OCC approach on resource-constrained devices should be considered. Finally, there is a need to validate the feasibility of implementing the proposed scheme in real-world scenarios.

## Figures and Tables

**Table 1:** A comparison performance of YOLOv8 family using our LED array detection dataset.

Model	Batch Size	mAP0.5:95	mAP0.5	Speed (ms)	Precision	GFLOPs	Weight
X	32	0.86817	0.999	4.566	0.999	258.133	136.7
	16	0.86744	0.99744	8.206	0.99744	258.133	136.7
	8	0.86744	0.99744	8.917	0.99744	258.133	136.7
L	32	0.86964	0.99498	2.825	0.999	165.412	87.6
	16	0.86936	0.995	3.103	0.99999	165.412	87.6
	8	0.86519	0.995	6.627	0.99959	165.412	87.6
M	32	0.86341	0.995	1.954	0.998	79.072	52
	16	0.86627	0.995	3.301	0.99799	79.072	52
	8	0.86634	0.99495	4.827	0.99725	79.072	52
S	32	0.86004	0.995	1.25	0.99	28.651	22.5
	16	0.86169	0.99497	1.834	0.999	28.651	22.5
	8	0.86453	0.995	2.536	0.997	28.651	22.5

**Table 2:** Performance comparison of YOLOv8 and YOLOv5 using our LED array detection dataset.

Model	mAP0.5:95	mAP0.5	Speed (ms)	Precision	GFLOPs	Weight
YOLOv8-X	0.86817	0.999	4.566	0.999	258.133	136.7
YOLOv5-X	0.87744	0.995	10.68	0.999	238.133	169.15
YOLOv8-L	0.86964	0.994	2.825	0.999	165.412	87.6
YOLOv5-L	0.85949	0.995	6.525	0.999	165.412	90.63
YOLOv8-M	0.86341	0.995	1.954	0.998	79.072	52
YOLOv5-M	0.84974	0.995	3.715	0.999	64.358	53.88
YOLOv8-S	0.86004	0.995	1.25	0.99	28.651	22.5
YOLOv5-S	0.85497	0.995	2.351	0.999	28.651	24.44

## Data Availability

Not applicable.
